# Trends in hyperlipidemia and hypertension management in type 2 diabetes patients from 1998–2004: a longitudinal observational study

**DOI:** 10.1186/1475-2840-6-25

**Published:** 2007-09-20

**Authors:** Jacoba P Greving, Petra Denig, Dick de Zeeuw, Henk JG Bilo, Flora M Haaijer-Ruskamp

**Affiliations:** 1 Department of Clinical Pharmacology, University Medical Center Groningen, University of Groningen, The Netherlands; 2 Department of Internal Medicine, Isala Clinics, Zwolle, The Netherlands

## Abstract

**Background:**

Lack of treatment initiation or intensification might explain why some patients with type 2 diabetes do not reach target goals. The objective is to assess trends in risk factor treatment, and identify determinants for medication adjustments in patients with uncontrolled hypertension and/or hyperlipidemia.

**Methods:**

We conducted a cohort study using data from the Zwolle Outpatient Diabetes project Integrated Available Care (ZODIAC)-study in The Netherlands. Management of hypertension and hyperlipidemia was assessed yearly from 1998–2004 by measuring the percentage of patients receiving a treatment initiation or intensification among all patients with elevated risk factor levels. Generalized estimating equation analyses were performed.

**Results:**

During the study period, the percentage of patients with an elevated total cholesterol/high-density lipoproteins ratio (>6) decreased considerably (from 29% to 4%) whereas the percentage of hypertensive patients decreased only slightly (≥ 150/85 mmHg; from 58% to 51%). Initiation of lipid-lowering therapy and intensification of antihypertensive therapy was higher in more recent years. However, still two-third of patients with insufficiently controlled blood pressure in 2003 did not receive an initiation or intensification of antihypertensive treatment in the following year. Treatment changes were mainly determined by elevated levels of the corresponding risk factor. We did not observe increased initiation rates for lipid-lowering therapy in patients with both hypertension and hyperlipidemia.

**Conclusion:**

Hypertension and hyperlipidemia management in type 2 diabetes patients has improved in the past decade but further improvement is possible. Greater effort is needed to stimulate medication adjustments in patients with insufficiently controlled hypertension and combined risk factors.

## Background

The increased incidence of cardiovascular disease (CVD) among patients with type 2 diabetes has led to increased recognition of hypertension and hyperlipidemia as important targets of therapy in addition to hyperglycemia [1,2]. Clinical trials in patients with type 2 diabetes convincingly demonstrated that cholesterol reduction and tight blood pressure control reduce the risk of major cardiovascular events [3-5]. Diabetes guidelines therefore advocate an intensified treatment approach aiming at all risk factors for the primary prevention of CVD [6-9].

It has been shown that although increasing numbers of diabetes mellitus patients are nowadays tested for relevant risk factors, much smaller percentages reach target goals [10-12]. These findings might be explained by low rates of medication initiation and dose adjustment in patients with elevated risk factor levels [11,13,14]. In addition, there are doubts that general practitioners have sufficiently implemented a multiple risk factor approach in routine practice [15,16]. This could also contribute to patients being undertreated. Observational studies so far, however, have focussed mainly on the influence of single elevated risk factors on treatment modifications [11,13,14]. Moreover, these studies have only looked at changes in drug regimes over short periods of time, not allowing for the assessment of trends. It is therefore not clear whether treatment of multiple risk factors in patients with diabetes has intensified over the past years.

The objectives of the present study were (1) to examine trends in initiation and intensification of lipid-lowering and antihypertensive drug therapy among type 2 diabetes patients, and (2) to analyze factors associated with these drug regime changes, in particular looking at combined risk factors.

## Methods

### Setting

This study was conducted as part of an ongoing longitudinal study, the Zwolle Outpatient Diabetes project Integrated Available Care (ZODIAC)-study in The Netherlands. The ZODIAC-study is a shared-care project for type 2 diabetes within the primary setting that started in 1998. Details about this project have been published previously [17]. In brief, general practitioners (GPs) are supported by diabetes specialist nurses (DSNs) for conducting the annual control of their type 2 diabetes patients. The GPs kept full responsibility for the care of these patients and remained responsible for drug prescribing and check-ups that should take place every three months. The number of participating GPs ranged from 32 in 1998 to 46 in 2004.

### Study subjects

The study population represents a dynamic cohort of type 2 diabetes patients who had at least two visits in consecutive years for their annual control to a DSN between 1998 and 2004. During this period, all patients with known and newly diagnosed type 2 diabetes were included when they met the following criteria in the judgement of their GP: (1) treated exclusively in primary care; (2) no terminal illness, and (3) no severe deficits in memory and understanding.

### Measurements

The annual visit to the DSN included registration of the following data: (1) medication use (general and diabetes medication) and medical history as provided by the GP and checked with the patient; (2) height, weight, blood pressure measured at the visit; and (3) laboratory values that had been measured before the visit. The laboratory measurements were glycosylated haemoglobin (HbA1c), total cholesterol (TC), high-density lipoproteins (HDL) and low-density lipoproteins (LDL). All laboratory measurements were performed in a central laboratory. Medical history included year of diabetes onset and history of myocardial infarction and/or angina pectoris. Body mass index was calculated from weight and length (kg/m^2^). The TC/HDL ratio was calculated from total cholesterol and HDL cholesterol.

### Guideline recommendations

During the study period, the Dutch General Practitioners' guidelines recommended a combined assessment of blood pressure and lipid levels to guide treatment to patients at high risk for CVD [6,18]. Lipid-lowering therapy should be targeted to patients at greatest risk for CVD: i.e. patients with pre-existing CVD, patients with a (suspected) hereditary lipid disorder or patients with an estimated 10-year coronary heart disease risk larger than 25% as based on the Framingham risk score [6,18]. To eliminate the need for GPs to calculate risk scores, the guidelines incorporate colour-coded risk tables to guide management for primary prevention based on a person's age, sex, smoking status, blood pressure, and TC/HDL ratio. From these tables, it can be derived that the presence of hypertension determines the need for lipid-lowering treatment in non-smoking patients with a TC/HDL ratio of 5–7, and in smoking patients with a TC/HDL ratio of 4–6. The guideline, however, also provides two simplified recommendations for the primary prevention: men aged 50–70 years and women aged 50–75 years should receive lipid-lowering therapy when their TC/HDL ratio is higher than 6 for non-smoking patients and when their TC/HDL ratio is higher than 5 for smoking patients [6]. Furthermore, patients with hypertension (defined as a systolic blood pressure (SBP) ≥ 150 mmHg or a diastolic blood pressure (DBP) ≥ 85 mmHg) should be treated with antihypertensive drugs [6,19].

### Changes in drug therapy

Using the prescribing information from reports of annual controls by the DSNs, we determined for each patient in each year whether the patient had received an initiation or intensification of drug therapy. Drug therapy was considered to have been intensified if the dose was increased or an additional drug class was added. A switch to another drug class without continuation of the original medication was not considered as an intensification of therapy, because patients could have been switched due to side effects. Trends in drug regime changes were studied over the period 1999–2004 to determine whether the GPs changed prescribing rates for lipid-lowering and antihypertensive drug therapy. Furthermore, we examined to what extent such changes in drug treatment were determined by risk factor levels measured prior to these changes.

### Statistical analyses

Descriptive analyses characterize the population of type 2 diabetes patients over time, and show the frequencies of drug regime initiations and intensifications in patients with elevated risk factor levels. To identify determinants for initiation and intensification of lipid-lowering and antihypertensive drug therapy, generalized estimating equation analyses were performed using STATA 8. With generalized estimating equation analysis, the relation between longitudinally measured variables can be studied correcting for within person correlations caused by the repeated measurements for one subject. Models were constructed for the changes in antihypertensive treatment and changes in lipid-lowering treatment, and for initiation and intensification separately. We assessed the influence of the following factors: age, gender, SBP, DBP, TC/HDL ratio and TC preceding a possible treatment change. Factors that contributed significantly (p < 0.05) to the model were maintained in the final model (forward stepwise regression). To test a possible combined effect of blood pressure and lipid levels, an interaction term of SBP with TC/HDL ratio was included in the models. We adjusted for HbA1c, diabetes duration, history of myocardial infarction and/or angina pectoris, body mass index, and year of screening. Because initiation of lipid-lowering treatment during our study period was recommended in men younger than 70 years and women younger than 75 years, we repeated analyses for lipid-lowering therapy including only these patients. Results are presented as odds ratios (OR) with corresponding confidence intervals (CI).

## Results

### Characteristics of the study cohort

The study population ranged from 946 to 1485 type 2 diabetes patients over the 6-year period from 1998 to 2003. The mean age changed slightly from 68 years in 1998 to 67 years in 2003. Women represented the majority (on average 57%) of the study population (Table 1).

**Table 1:** Characteristics of type 2 diabetes patients

	**1998** N = 946	**1999** N = 1075	**2000** N = 1248	**2001** N = 1374	**2002** N = 1295	**2003** N = 1485
***Patient characteristics***						
Age (years)	68 ± 11	68 ± 11	67 ± 11	67 ± 11	67 ± 11	67 ± 11
Female sex (%)	57%	58%	57%	57%	57%	55%
Duration of diabetes (years)	5 (2–10)	5 (2–10)	5 (2–10)	4 (2–9)	4 (2–9)	5 (2–9)
History of MI/AP	25%	22%	21%	19%	18%	18%
Body mass index (kg/m^2^)	29.0 ± 4.7	29.0 ± 4.7	29.4 ± 4.8	29.5 ± 4.8	29.5 ± 4.7	29.5 ± 4.8
HbA1c (% units)	7.5 ± 1.2	7.4 ± 1.2	7.3 ± 1.3	7.0 ± 1.2	7.0 ± 1.2	7.0 ± 1.2
TC/HDL ratio	5.3 ± 1.6	4.8 ± 1.3	4.5 ± 1.2	4.4 ± 1.2	4.1 ± 1.1	3.9 ± 1.1
Systolic blood pressure (mmHg)	155 ± 25	150 ± 23	150 ± 23	146 ± 20	145 ± 21	148 ± 21
Diastolic blood pressure (mmHg)	84 ± 11	82 ± 11	81 ± 11	80 ± 10	80 ± 10	84 ± 11
Number of glucose-lowering drugs						
None	20%	17%	18%	20%	23%	20%
1 oral	43%	43%	41%	39%	36%	39%
≥ 2 oral	22%	25%	26%	26%	28%	26%
Insulin (with or without oral drugs)	15%	15%	15%	15%	13%	14%
Use of cardiovascular drugs						
Lipid-lowering drugs	12%	15%	22%	27%	30%	34%
Antihypertensive drugs	48%	51%	57%	63%	66%	69%
ACE-inhibitors or ARBs	24%	26%	30%	36%	42%	45%
Antiplatelet drugs	22%	22%	23%	25%	25%	26%

Values are percentages, means ± standard deviations or median (interquartile range).

MI, myocardial infarction; AP, angina pectoris; HbA1c, haemoglobin A1c; TC, total cholesterol; HDL, high-density lipoprotein; ACE-inhibitors, angiotensin-converting enzyme inhibitors; ARBs, angiotensin II receptor blockers.

The median duration of diabetes was 5 years and remained reasonably stable over the years. Overall, 65% of the patients was treated with oral hypoglycaemic drugs only, and 15% received a combination of oral hypoglycaemic drugs and insulin or insulin alone. We observed an increase in the use of lipid-lowering drug treatments (from 12% to 34%) and antihypertensive drug treatments (from 48% to 69%) and substantial decreases in mean HbA1c, TC/HDL ratio, and systolic blood pressure values between 1998 and 2003 (Table 1).

### Management of hyperlipidemia

**Figure 1:**
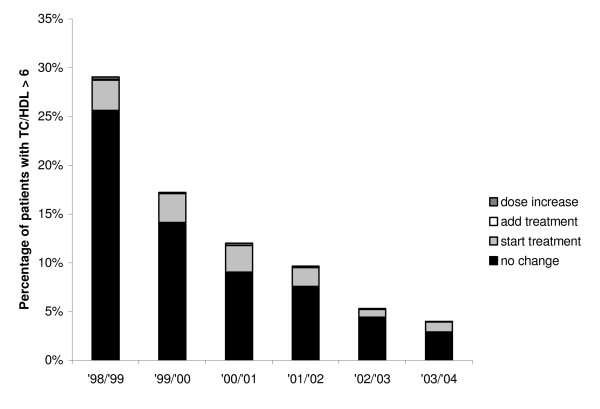
**Trends in percentages of patients with elevated TC/HDL values and subsequent treatment modifications (1998–2004)**. TC, total cholesterol; HDL, high-density lipoprotein.

During the study period, the percentage of patients with an elevated TC/HDL ratio (>6) decreased considerably from 29% (273/939) to 4% (59/1471) (Figure 1). In 1998, 9% (25/273) of these insufficiently controlled patients were already on lipid-lowering drug therapy, and 11% (29/273) started treatment in the following year. By 2003, these percentages had increased to 25% (15/59) on drug therapy, and another 26% (15/58) starting treatment in the subsequent year. Almost no dose increase or addition of a drug class occurred over the years (Figure 1).

**Figure 2:**
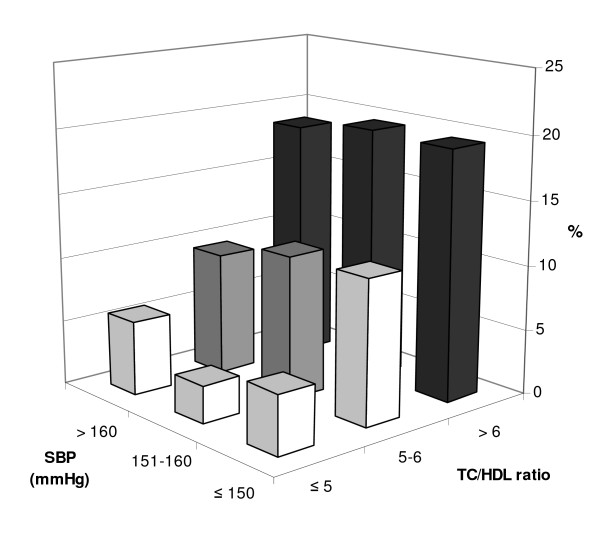
**Percentage of patients in subgroups stratified by lipid and blood pressure levels initiated on lipid-lowering therapy**. Black bars = lipid-lowering therapy recommended for most patients aged 50–70; grey bars = lipid-lowering therapy recommended for most smoking patients and males aged 60–70 years; white bars = lipid-lowering therapy seldom recommended. SBP, systolic blood pressure; TC, total cholesterol; HDL, high-density lipoprotein.

In multivariable analyses, TC/HDL ratio, SBP, age, history of myocardial infarction and/or angina pectoris, and year of screening were predictors of subsequent initiation of lipid-lowering therapy, while gender was not found to be associated with treatment initiation (Table 2). There was no significant interaction between TC/HDL values and SBP levels, suggesting that the association between TC/HDL ratio and subsequent initiation of lipid-lowering therapy does not differ by SBP levels. Accordingly, we observed no difference in the proportion of patients initiated on lipid-lowering therapy who had elevated SBP levels compared to normal SBP levels (Figure 2). Only 9% of patients with SBP levels above 160 mmHg and TC/HDL values between 5 and 6 were receiving a treatment initiation, which was not higher than for patients with similar TC/HDL values and SBP levels below 150 mmHg.

**Table 2:** Multivariable analyses of factors associated with initiation and intensification of lipid-lowering and antihypertensive drug therapy

	***Lipid-lowering drug therapy***		***Antihypertensive drug therapy***	
	
	***Initiation***	***Intensification***	***Initiation***	***Intensification***
	OR*(95% CI)	OR(95% CI)	OR(95% CI)	OR(95% CI)
Age (per 10 years)	0.7 (0.6–0.8)	-	1.2 (1.1–1.3)	1.1 (1.0–1.2)
Gender	-	-	-	-
HbA1c (%)	-	-	0.9 (0.8–1.0)	-
Systolic blood pressure (per 10 mmHg)	1.1 (1.0–1.1)	-	1.3 (1.2–1.4)	1.1 (1.1–1.2)
Diastolic blood pressure (per 10 mmHg)	-	-	1.2 (1.0–1.4)	1.1 (1.0–1.2)
TC/HDL ratio	1.8 (1.6–1.9)	1.3 (1.1–1.5)	1.2 (1.1–1.3)	-
History of MI/AP	1.9 (1.4–2.5)	-	2.3 (1.6–3.3)	-
Year of screening	1.3 (1.2–1.4)	0.9 (0.8–1.0)	1.1 (1.1–1.2)	1.1(1.1–1.1)

* Additionally adjusted for body mass index. OR, odds ratio; HbA1c, haemoglobin A1c; TC, total cholesterol; HDL, high-density lipoprotein; MI, myocardial infarction; AP, angina pectoris.

Intensification of lipid-lowering therapy was associated with TC/HDL ratio and year of screening, but not with SBP or other risk factors (Table 2). In subgroup analyses of younger patients (men aged 70 years or younger and women aged 75 years or younger), we found similar point estimates for initiation and intensification of lipid-lowering therapy, but age and SBP were not statistically significant determinants anymore. Using total cholesterol as risk factor instead of TC/HDL ratio yielded similar results (data not shown).

### Management of hypertension

**Figure 3:**
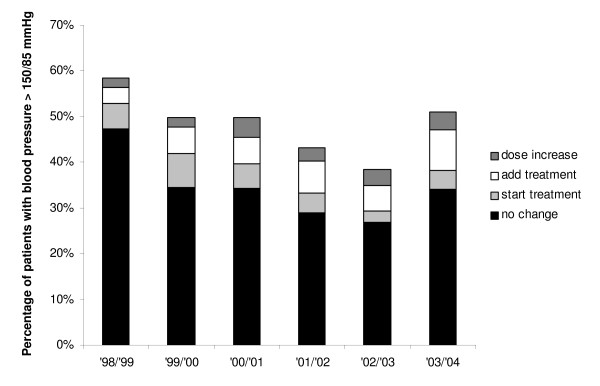
Trends in percentages of patients with elevated blood pressure levels and subsequent treatment modifications (1998–2004).

The percentage of hypertensive patients (SBP ≥ 150 mmHg or DBP ≥ 85 mmHg) decreased from 58% (544/930) in 1998 to 51% (754/1479) in 2003 (Figure 3). In 1998, 51% (279/544) of the patients not reaching blood pressure targets were already on antihypertensive drug therapy, and 10% (52/541) started treatment in the following year. By 2003, the percentage already treated had increased to 72% (543/754), and another 8% of patients started antihypertensive therapy (61/748). In the uncontrolled patients already on treatment, 19% (52/276) received an intensification in 1999 and 35% (186/539) in 2004. Adding drugs was more common than increasing the dose (Figure 3). Treatment intensifications were less likely to occur in patients taking already two antihypertensive drugs in 1998, and three or more drugs in 2003 (data not shown). The percentage of uncontrolled patients receiving no change in treatment decreased from 81% in 1998 to 67% in 2003 (black proportion of the bars in Figure 3).

In multivariable analyses, initiation of antihypertensive therapy was positively related to preceding levels of SBP, DBP, TC/HDL ratio, age, history of myocardial infarction and/or angina pectoris, and year of screening, and negatively related to HbA1c. Intensification of antihypertensive drug therapy was positively related to SBP, DBP, age, and year of screening, but not with TC/HDL ratio or other risk factors (Table 2).

## Discussion

In this large observational study, we observed an overall increased use of antihypertensive and lipid-lowering drugs, and better control of risk factors between 1998 and 2004. For lipid-lowering treatment, improvements were mainly due to an increased proportion of type 2 diabetes patients who were initiated on drug treatment, whereas for hypertension improvements especially concerned the intensification of treatment in patients already on antihypertensive therapy. In general, treatment initiations were more likely in patients with related cardiovascular comorbidity. Otherwise, treatment changes were mainly determined by elevated levels of the corresponding risk factor. We did not observe increased initiation rates of drug therapy in patients with both hypertension and hyperlipidemia. We also did not observe any gender differences regarding the initiation or intensification of drug treatment.

Despite these generally favourable improvements in the management of hyperlipidemia and hypertension, still only one-third of patients with insufficiently controlled blood pressure or lipid ratio levels in 2003 received an initiation or intensification of antihypertensive or lipid-lowering treatment in 2004. Other studies have reported similar low rates of treatment initiations, but higher rates of intensifications particularly for lipid-lowering therapy [11,20,21]. In a study in an US academic medical centre in 1997 to 1999, 30% with elevated LDL cholesterol levels received a treatment intensification, and 30% of patients with elevated SBP levels [21]. In a Veterans Affairs study in 1998 to 1999, 39% of patients with diabetes and elevated LDL cholesterol levels received either a treatment initiation or intensification [20]. More recently, a study within a US medical care program in 2002 to 2003 showed even higher rates of therapy initiation or intensification: 64% of patients with insufficiently controlled SBP levels and 47% for insufficiently controlled LDL cholesterol levels [22]. However, their study population included all patients with hypertension, hyperlipidemia, diabetes mellitus or a combination of these conditions. It is known that patients with diabetes receive less intensive antihypertensive and lipid-lowering medication therapy than patients without diabetes [14]. Also, it should be noted that in our study, the number of patients with insufficiently controlled lipid levels was quite low in the latter years, leaving not much room for further improvement in this group of patients. The lack of gender differences suggests that disparities in quality of care measures observed in other countries regarding cardiovascular disease and diabetes management may not be that distinct in The Netherlands [23].

Several causes have been proposed why physicians may not initiate or intensify therapy [24]. Physicians may be reluctant to prescribe additional drugs because of concerns about medicalisation and poor adherence to treatment [25-27]. At the beginning of our study period, GPs were clearly less eager to intensify antihypertensive treatment in patients already receiving two or more antihypertensive drugs. Secondly, there may be barriers related to the strict high-risk approach to primary prevention. Aggressive treatment may not be beneficial or safe for all patients [28]. Although we used relatively lenient target levels for both blood pressure and lipid control in our study, some GPs may accept higher levels, especially in patient without additional cardiovascular comorbidity. It has also been suggested that the combined risk approach may be difficult to implement in routine practice. The content and format of cardiovascular risk tables have been criticized, and risk calculator use is not common [15,16]. We indeed observed that the percentage of lipid-lowering treatment initiations was not higher in patients with elevated blood pressure levels. According to guideline recommendations, lipid-lowering therapy is indicated in patients with high lipid ratio levels but also in patient with lower lipid ratio levels who have hypertension. Results of multivariable analyses showed that TC/HDL ratio was the strongest predictor of the initiation of lipid-lowering therapy and SBP levels only had a weak effect. More importantly, there was no significant interaction between TC/HDL values and SBP levels, which suggests that the recommended combined assessment of blood pressure and lipid levels has not yet been adopted in clinical practice. Our finding that physicians primarily manage single risk factors is consistent with results from other recent studies [11,29-31].

A limitation of this study is that we evaluated the management of hyperlipidemia and hypertension within a shared-care project. Specialist nurses performed the annual control of type 2 diabetes patients which may have facilitated physicians to provide better care [17]. Specialist nurses for diabetes care are, however, common in many health care settings. Another limitation is that the data were collected on an annual basis. As a result we could not assess whether physicians responded immediately to a visit of an elevated risk factor level. Since many patients with insufficiently controlled blood pressure or lipid ratio levels did not receive a treatment initiation or intensification in the following year, our results suggests that physicians missed several opportunities to increase medication regimes or dosage. An important strength of this study is that data were collected over a long time period enabling to assess trends in treatment initiation and intensification over a 6-year period. The average demographic characteristics and diabetes duration in our study population remained stable over these years.

## Conclusion

Although our study shows that the management of hypertension and hyperlipidemia in patients with diabetes has improved in the past decade, it also demonstrates specific problems that need more attention. Physicians' readiness to start lipid-lowering therapy in patients with diabetes has increased but awareness for more intensive treatment in patients with additional hypertension appears to be lacking. In addition, the willingness to intensify antihypertensive treatment in patients with elevated blood pressure levels remains low. In the majority of treated patients no further treatment changes were made, and dosages were seldom increased despite uncontrolled risk factor levels.

## List of abbreviations used

ACE-inhibitors- Angiotensin-converting enzyme inhibitors;

AP- Angina pectoris;

ARBs- Angiotensin II receptor blockers;

CI- Confidence intervals;

CVD- Cardiovascular disease;

DBP- Diastolic blood pressure;

DSNs- Diabetes specialist nurses;

GPs- General practitioners;

HbA1c- Haemoglobin A1c;

HDL- High-density lipoprotein;

LDL- Low-density lipoprotein;

MI- Myocardial infarction;

OR- Odds ratio;

SBP- Systolic blood pressure;

TC- Total cholesterol;

US- United States;

ZODIAC- Zwolle Outpatient Diabetes project Integrated Available Care.

## Competing interests

The authors declare that they have no competing interests.

## Authors' contributions

JPG and PD designed the study. JPG performed the statistical analysis. PD, DdZ, HJGB and FMH helped to interpret the results. HJGB was involved in the establishment of the database used in this study. JPG and PD led the writing of this manuscript but all listed authors contributed substantially to the editorial process and approved the final manuscript.
